# Neonatal appendicitis mimicking intestinal duplication: a case report

**DOI:** 10.1186/1752-1947-6-286

**Published:** 2012-09-10

**Authors:** Isamu Saeki, Takeshi Yamanouchi, Sakura Tanaka, Takashi Kawanami, Ryou Mori, Yoshio Zaizen

**Affiliations:** 1 Department of Pediatric Surgery, Fukuoka Children’s Hospital and Medical Center for Infectious Diseases, 2-5-1 Tojin-machi Chuo-ku, Fukuoka, 810-0063, Japan; 2 Department of Radiology, Fukuoka Children’s Hospital and Medical Center for Infectious Diseases, 2-5-1 Tojin-machi Chuo-ku, Fukuoka, 810-0063, Japan

## Abstract

**Introduction:**

Acute appendicitis is a common disease in older children but rare in neonates.

**Case presentation:**

We report the case of a 2-day-old Asian baby who suffered from neonatal appendicitis mimicking intestinal duplication. Laparoscopic appendectomy was successfully performed after the trans-umbilical division of adhesions, and the postoperative course was uneventful.

**Conclusion:**

There are few reports describing abdominal masses caused by appendicitis mimicking intestinal duplication. The laparoscopic approach for neonatal appendicitis is considered to be a safe and useful therapeutic modality with good cosmetic results.

## Introduction

Neonatal appendicitis is rare and has a high mortality rate [1-3]. Diagnosis is difficult and sometimes necrotizing enterocolitis (NEC) or focal intestinal perforation (FIP) are suspected [4,5]. We present a case of neonatal appendicitis that presented as an abdominal mass mimicking intestinal duplication before surgery. The trans-umbilical division of adhesion and laparoscopic surgery revealed a diagnosis of appendicitis and laparoscopic appendectomy was successfully performed.

## Case presentation

A 2-day-old Asian baby, born at 36 weeks’ gestation, weighing 2944g, was admitted to a local hospital for abdominal distension and poor general status. His white blood cell count was 19,800 (Neutrophil 75%). The administration of antibiotics and non-per oral feeding were performed based on a preliminary diagnosis of sepsis and paralytic ileus. His condition improved slightly; however, he was admitted to our hospital on the fourth day after his birth under a suspicion of NEC. His abdomen was slightly distended but a normal color. The results of ultrasonography examinations and an X-ray showed no signs of NEC (i.e., there was no evidence of free air, peritoneal effusion or peritonitis). A lower intestinal tract contrast study showed a normal colon structure. The results of a spinal fluid test revealed an increased cell count, and conservative management was continued under suspicion of sepsis and meningitis.

**Figure 1:**
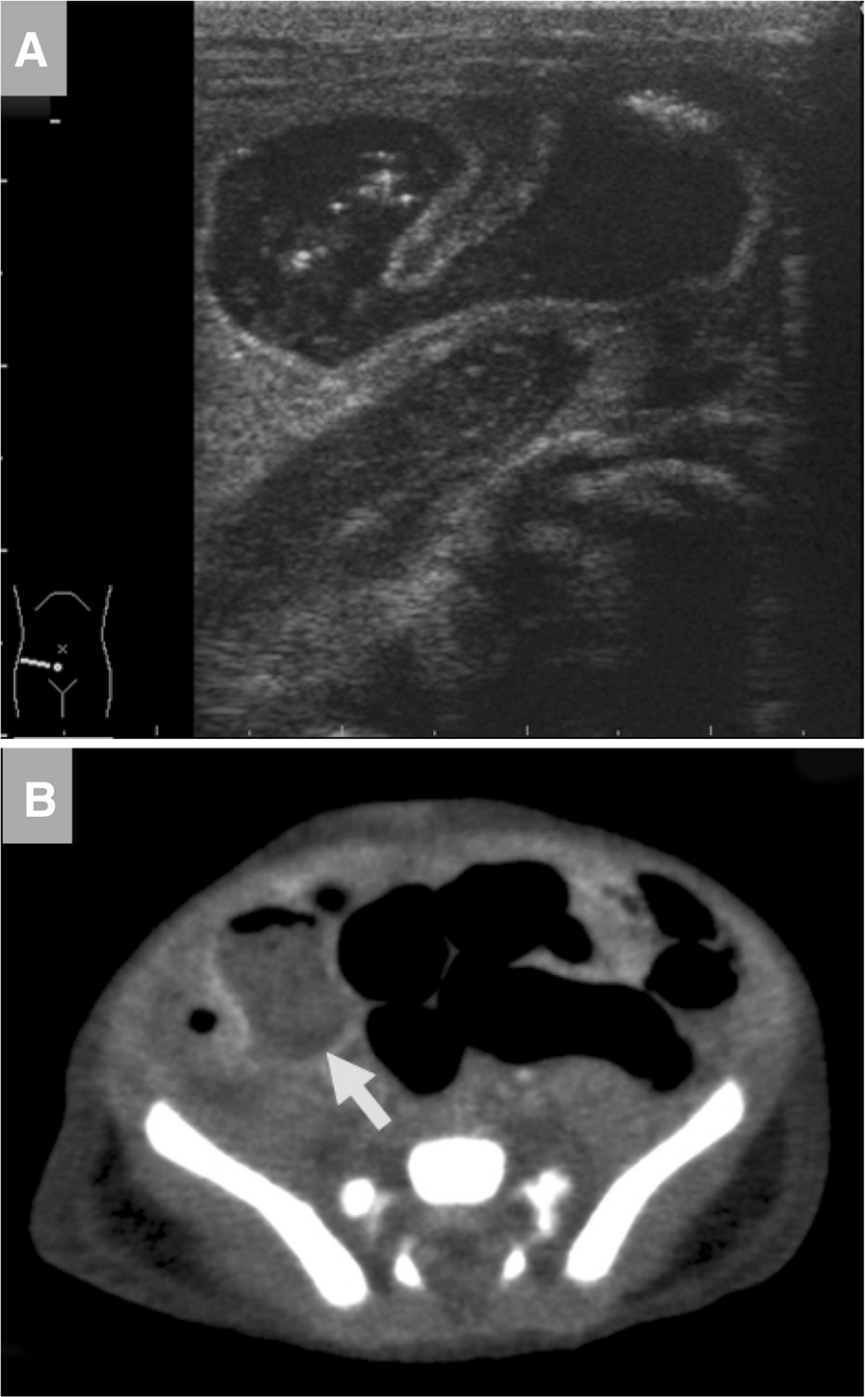
**Ultrasonography and computed tomography images.A**) Ultrasonography shows an intra-abdominal cyst with a double layer surrounding sign in the right lower abdomen. **B**) Computed tomography shows a cyst with an enhanced wall (Arrow).

An abdominal ultrasonography examination 6 days after birth showed an abdominal cyst with a double layer surrounding sign at right lower quadrant (Figure 1A). Computed tomography showed a cyst with an enhanced wall in the area and the intestine had a normal wall thickness (Figure 1B). His inflammation improved quickly and at 10 days after birth his abdomen was slightly distended but soft, and it also demonstrated normal intestinal sounds before surgery. Intestinal duplication was diagnosed and he underwent surgery.

**Figure 2:**
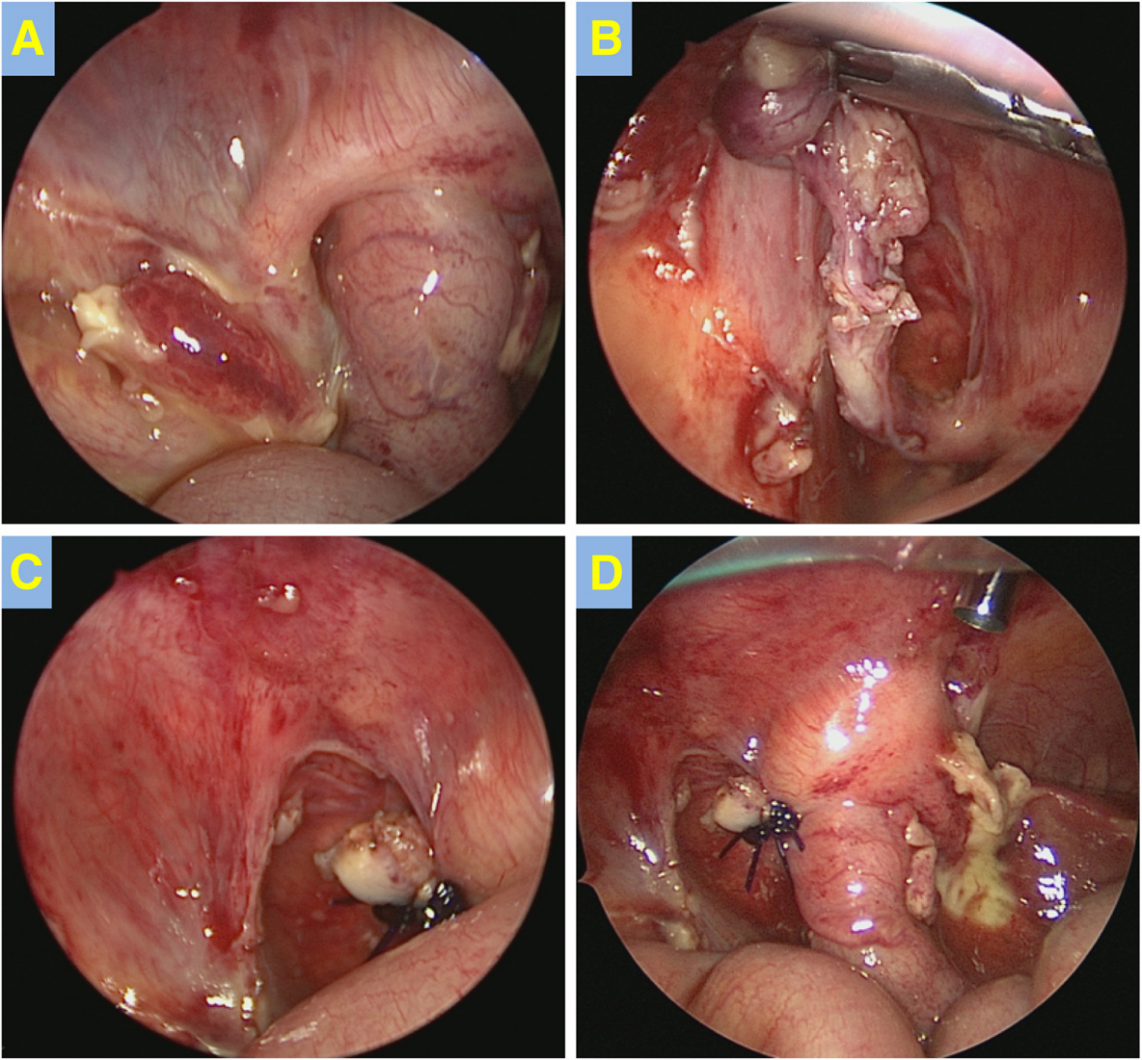
**Surgical findings.A**) Appendix was inflamed and adhesive. (**B**,**C**) Laparoscopic appendectomy was performed. **D**) Abscess and strong adhesions were recognized around the ascending colon.

**Figure 3:**
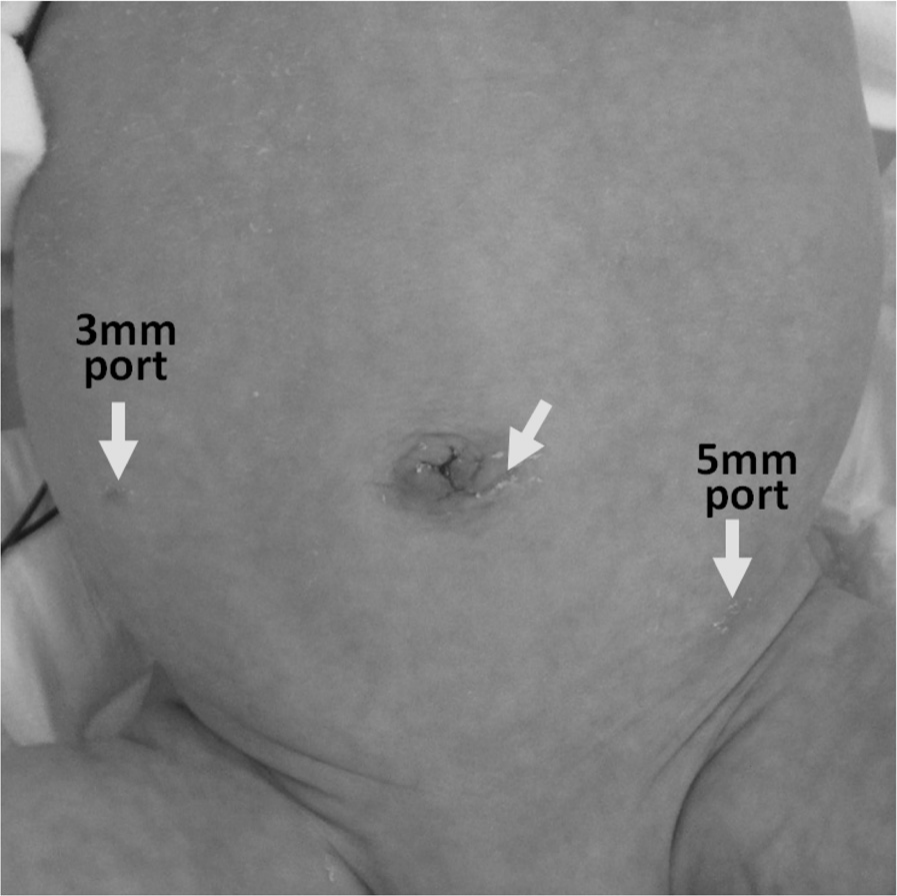
**Wound scar 7 days after surgery.** Incision sites are indicated with arrows.

The open Hasson technique was used. A 5mm umbilical port was inserted via an inferior-umbilical incision. The intestine, colon and peritoneum all strongly adhered to the right abdominal region and no other ports could be inserted. A wound retractor XS™ (Applied Medical, Rancho Santa Margarita, CA, USA) was used to perform the trans-umbilical division of any adhesions. Laparoscopic examination revealed adhesions and inflammation around the ileum, ascending colon and liver bed and an inflamed appendix was strongly attached to the retro-peritoneum (Figures 2A–D). No intestinal duplication was observed. The surgical findings included perforated appendicitis and peritonitis. The abdominal cyst identified preoperatively was thought to be an inflamed ascending colon. A trans-umbilical operation was impossible due to strong adhesion around the patient’s appendix. Into the right lateral abdomen 3mm instruments were inserted and 5mm instruments were inserted into the left lower abdomen (Figure 3). The adhesions and mesoappendix were divided using SonoSurg™ (Olympus, Tokyo, Japan) and laparoscopic appendectomy was then successfully performed. The patient started feeding 2 days after surgery and his postoperative clinical course was uneventful.

## Discussion

Although acute appendicitis is a very common disease in adults and children, the incidence of neonatal appendicitis is rare [1-3]. A male dominance (75%) has been reported [3]. Although there has been a significant reduction of the mortality rate over the past decade, the mortality rate is still high at 28% [3].

The causes of neonatal appendicitis are thought to differ from those in adults and older children. Amyand’s hernia and other coexisting diseases (e.g. Hirschsprung’s disease and cystic fibrosis) may cause appendicitis [6,7]. Prematurity or other severe conditions may cause vascular insufficiency and perforation of the appendix [2,3]. NEC is localized to the appendix [1]. However, in the current case, there were no coexisting diseases or any severe conditions that might have induced poor circulation before the onset of symptoms.

Neonatal appendicitis has no specific signs or symptoms, therefore a preoperative diagnosis is very difficult and the majority of neonates have so far been diagnosed intra-operatively [2]. A differential diagnosis in such cases must be made for such diseases as NEC, FIP, Hirschsprung’s disease or midgut volvulus. Unfortunately, there are few reports describing abdominal masses caused by appendicitis mimicking intestinal duplication. We also could not diagnose this case preoperatively. In such a situation, a trans-umbilical examination using laparoscopy is useful, even in neonates. Even if there are strong intra-abdominal adhesions, the trans-umbilical division of such adhesions and sufficient laparoscopic examinations can be easily performed.

Laparoscopic appendectomy was performed on the neonate. This is the third case (second report) of laparoscopic appendectomy for neonatal appendicitis [8]. Tajiri *et al*. [9] reported using a trans-umbilical approach for various neonatal surgical diseases, which is woundless with only an umbilical incision, but this method is not available in cases of severe abdominal adhesions, as observed in the current case. The laparoscopic approach for neonatal appendicitis is therefore considered to be a safe and useful therapeutic modality with good cosmetic results.

## Conclusion

There are few reports describing abdominal masses caused by appendicitis mimicking intestinal duplication. The laparoscopic approach for neonatal appendicitis is considered to be a safe and useful therapeutic modality with good cosmetic results.

## Consent

Written informed consent was obtained from the parents of the patient for publication of this case report and accompanying images. A copy of the written consent is available for review by the Editor-in-Chief of this journal.

## Abbreviations

CT, computed tomography; FIP, focal intestinal perforation; NEC, necrotizing enterocolitis.

## Competing interests

The authors declare that they have no competing interests.

## Authors’ contributions

IS, TY and RM were operators of this case study. ST and YZ were major contributors to the editing and revising of the manuscript. TK was a contributor in evaluating the radiological aspects of the manuscript. All authors read and reviewed the final manuscript.
